# Defined Intestinal Regions Are Drained by Specific Lymph Nodes That Mount Distinct Th1 and Th2 Responses Against *Schistosoma mansoni* Eggs

**DOI:** 10.3389/fimmu.2020.592325

**Published:** 2020-10-23

**Authors:** Johannes U. Mayer, Sheila L. Brown, Andrew S. MacDonald, Simon W. Milling

**Affiliations:** ^1^Centre for Immunobiology, Institute of Infection, Immunity and Inflammation, College of Medical, Veterinary & Life Sciences, University of Glasgow, Glasgow, United Kingdom; ^2^Malaghan Institute of Medical Research, Wellington, New Zealand; ^3^Lydia Becker Institute of Immunology and Inflammation, Manchester Collaborative Centre for Inflammation Research, Faculty of Biology, Medicine and Health, Manchester Academic Health Science Centre, University of Manchester, Manchester, United Kingdom

**Keywords:** mucosal immunology, th1/th2 balance, helminth antigen, mesenteric lymph node, microsurgery, dendritic cells, *Schistosoma mansoni*, *Heligmosomoides polygyrus bakeri*

## Abstract

The balance of type 1 and type 2 immune responses plays a crucial role in anti-helminth immunity and can either support chronic infection or drive type 2 mediated expulsion of the parasite. Helminth antigens and secreted molecules directly influence this balance and induce a favorable immunological environment for the parasite’s survival. However, less is known if the site of infection also influences the balance of type 1 and type 2 immunity. Here, we report that tissue-specific immune responses are mounted against helminth antigens, which elicited strong IL-4 responses when injected into the skin, while the same antigen, delivered into the intestinal subserosa, induced increased IFN-*γ* and reduced Th2 responses. Immune responses in individual mesenteric lymph nodes that drain defined regions of the intestine furthermore displayed a site-specific pattern of type 1 and type 2 immunity after *Schistosoma mansoni* or *Heligmosomoides polygyrus* infection. *S. mansoni* egg-specific Th2 responses were detectable in all mesenteric lymph nodes but Th1 responses were only present in those draining the colon, while *H. polygyrus* infection elicited mixed Th1 and Th2 responses in the lymph nodes associated with the site of infection. Similar site-specific type 1 and type 2 immune responses were observed in the draining lymph nodes after the controlled delivery of *S. mansoni* eggs into different segments of the small and large intestine using microsurgical techniques. Different subsets of intestinal dendritic cells were hereby responsible for the uptake and priming of Th1 and Th2 responses against helminth antigens. Migratory CD11b^+^CD103^−^ and especially CD11b^+^CD103^+^ DC2s transported *S. mansoni* egg antigens to the draining lymph nodes to induce Th1 and Th2 responses, while CD103^+^ DC1s induced only IFN-*γ* responses. In contrast, *H. polygyrus* antigens were predominantly transported by CD11b^+^CD103^−^ DC2s and CD103^+^ DC1s and all DC subsets induced similar Th1 but weaker Th2 responses, compared to *S. mansoni* egg antigens. The development of adaptive anti-helminth immune responses is therefore influenced by the antigen itself, the uptake and priming characteristics of antigen-positive dendritic cell subsets and the site of infection, which shape the level of Th1 and Th2 responses in order to create a favorable immunological environment for the parasite.

## Introduction

The balance of different types of immune responses plays an important role in orchestrating the optimal immunological environment to appropriately counter infections. Numerous studies [reviewed in (1–4)] show that this is particularly relevant in the context of helminth infections, where type 2-biased immune responses promote parasite killing and worm expulsion, whereas type 1 immunity usually results in ineffective responses and chronic infection.

This effect is perhaps best demonstrated during the infection of the nematode *Trichuris muris* where Interleukin-4 (IL-4) and IL-13 mediated Th2 responses lead to rapid expulsion in resistant mouse strains, whereas susceptible mouse strains produce high levels of Interferon-gamma (IFN-*γ*), IL-12 and IL-18, which are characteristic of a predominant Th1 response (5, 6). IFN-*γ* depletion in normally susceptible animals furthermore promotes the development of protective Th2 responses, which renders these animals resistant to infection (7). Similarly, *T. muris*-resistant mice treated with IL-12 develop chronic infection (8), with similar observations made during *Nippostrongylus brasiliensis* infection, where parasitic worm expulsion is delayed after the injection of recombinant IL-12 (9). A direct requirement for IL-4/IL-13 signaling in worm expulsion is demonstrated by IL-4 receptor antibody blocking experiments, and in IL-4R^−/−^ and STAT6^−/−^ mice, dramatically limiting worm expulsion during *T. muris* (7), *Heligmosomoides polygyrus* (10) or *N. brasiliensis* (11) infection.

Th1 and Th2 responses also play an important role in *Schistosoma mansoni* infection, which afflicts over 200 million people worldwide (12). In humans, acute schistosomiasis manifests as an incapacitating febrile illness through the production of pro-inflammatory cytokines (13). With the onset of egg production by the female parasites a Th2-driven immune response is initiated, resulting in a mixed Th1/Th2 response which leads to chronic infection and granuloma formation in the liver and intestines (13, 14). In murine infection models, low levels of Th1/2 responses against developing worms become dominated by the potent type 2 immune response that develops against *S. mansoni* eggs, limiting damage to the host (15). In the absence of these Th2 responses, IL-4^−/−^ mice infected with *S. mansoni* experience severe TNF-α-mediated acute cachexia, hepatotoxicity, and high mortality (16), as well as intestinal pathology and detectable serum levels of LPS (17). Through experimental immunization models, *S. mansoni* eggs have been shown to induce both Th1 and Th2 responses in an antigen-specific manner (18, 19), and several antigens that can initiate and influence Th2 responses have been identified to date (20–23). An assessment of the ratios of type 1 and type 2 immune responses induced by the injection of *S. mansoni* eggs, however, suggests that other factors such as the different routes of immunization can influence anti-parasite immunity (18, 24, 25).

The idea that the tissue environment can influence T cell immunity has been well studied for tissue resident memory cells, which display tissue-specific signatures and functions (26–29). While tissue resident memory T cells acquire most of these characteristics after priming, a location-dependent preferential development of effector T cells has recently been identified in the intestinal draining lymph nodes (LNs) (30). The analysis of ROR*γ*t^+^ Th17 and Foxp3^+^ T regulatory cell development after controlled antigen delivery into different segments of the intestine revealed that tolerogenic responses preferentially develop in LNs draining the proximal intestine while Th17 responses are more pronounced in the LNs draining the distal intestine (30). This observation demonstrated that effector T cell differentiation can directly be influenced by location-specific factors, suggesting that the distinct phenotypes and proportions of pro-inflammatory and T regulatory cells, reported in numerous tissues and LNs (31–33), might not only be maintained but also initiated by location-specific cues.

Here we report that the site of immunization can indeed influence the balance of Th1 and Th2 responses against helminth antigens in a tissue- and site-specific manner. After immunization with *S. mansoni* eggs or *H. polygyrus* antigens in the footpad or the intestinal subserosa, antigen-specific CD4^+^ T cell responses in the lymph nodes showed a contrasting Th1/Th2 profile with higher IFN-*γ* responses observed after intestinal immunization, compared to increased Th2 responses after immunization of the skin. Distinct type 1 and type 2 immune responses were also observed in the individual mesenteric lymph nodes during live parasite infection, and stronger Th2 responses were observed in the LNs draining the proximal intestine compared to distal intestine. Similar observations were made after the controlled delivery of *S. mansoni* eggs into different segments of the small and large intestine, which induced increased Th1 and reduced Th2 responses in LNs draining the lower colon compared to the small intestine. Different helminth antigens were furthermore taken up by distinct subsets of intestinal migratory dendritic cells, which induced distinct levels of Th1 and Th2 responses, indicating that lymph node-specific type 1 and type 2 immune responses against helminth antigens are modulated by the antigen itself, antigen-positive dendritic cells and the site of infection.

## Materials and Methods

### Mice

C57BL/6 were obtained from Envigo, UK or bred at the University of Manchester, UK or the Malaghan Institute of Medical Research, Wellington, New Zealand. Mice were housed under specific pathogen free conditions and age- and gender-matched adult animals were used in each individual experiment.

### Surgical Procedures

All surgical procedures were carried out under aseptic conditions using inhalation anesthesia with Isoflurane (Abbot Animal Health). Agents, diluted in a maximum volume of 20 µl, were injected into the footpad using Micro-Fine Plus Hypodermic Syringes (29 G × 12.7 mm; BD Bioscience). To display the murine intestine and the mesenteric lymph nodes laparotomy surgery was performed. For lymph cannulations and MLN subcapsular injection, mice were gavaged with 0.2 ml Oliver oil 30 min prior to surgery to visualize the lymphatics. Buprenorphine (0.1 mg/kg; Vetergesic, Reckitt Benckiser Healthcare) and Carprofen (5 mg/kg; Rimadyl, Pfizer) were given subcutaneously into the flank as prophylactic analgesics. The abdominal area was shaved and sterilized, and the mouse placed on its back. A small incision into the skin of the animal’s midline was made using a scalpel. This incision was extended to up to 3 cm using scissors. Similarly, the muscle layer was incised at the midline, and the incision extended with scissors. The intestine was carefully displayed onto a moistened surgical cloth using cotton buds as shown in **Figure 2A**. Organs were moistened every 2–5 min during surgery to prevent them from drying out. Using a Leica M651 surgical microscope, agents, diluted in a maximum volume of 10 µl, were injected into the subserosal layer of the different segments of the intestine or under the MLN capsule using Micro-Fine Plus Hypodermic Syringes (29G × 12.7 mm; BD Bioscience). The intestinal segment of interest was gently held in place with forceps, and the needle was horizontally inserted with the bevel facing upwards. A small injection pocket would form at the injection site confirming that the intestinal lumen was not penetrated. For non-recovery ink injection experiments 10 μl of ink was injected into the intestinal subserosa and lymphatic drainage visualized using a Leica M651 surgical microscope (6×) with an attached Nikon camera. Different dyes were injected, which resulted in the following observations.

**Table:** 

Injected material	Observations
2% Chicago Blue dye (Sigma)	Wide-spread deposition and drainage *via* the lymphatics within minutes
2% Evans Blue dye (Sigma)	Wide-spread deposition and drainage *via* the lymphatics within minutes
Graphite particles (<20 μm, Sigma) resuspended in PBS	Very localized deposition but no lymphatic drainage
Black Chinese calligraphy ink (AMI)	Local deposition and drainage *via* the lymphatics within seconds
Black India ink (Pelikan)	Local deposition but no lymphatic drainage

After injection, the intestines were replaced into the body cavity, the muscle layer was sutured with 6.0 Vicryl absorbable sutures (Johnson and Johnson) using discontinuous stitching, and the skin was closed using surgical clips (Autoclip Wound Clip System, Harvard Apparatus). Mice were closely monitored post-surgery to ensure full recovery from the anesthesia and monitored on a daily basis thereafter. To collect lymph migrating DCs, MLNs were removed from 6-week-old male mice by laparotomy and blunt dissection, as previously described (25, 34). After 6 weeks, the thoracic lymph duct was cannulated by the insertion of a polyurethane medical grade intravascular tube (2Fr; Linton Instrumentation) and fluorescently labeled soluble parasite antigens were injected in the intestinal serosa. Lymph was collected for 18 h on ice in PBS (Gibco) supplemented with 20 U/ml of heparin sodium (Wockhardt UK).

### *Schistosoma mansoni* Infection

Mice were infected percutaneously with ~180 *S. mansoni* cercariae. Seven weeks after infection MLNs were collected. Infective *S. mansoni* cercariae were obtained from infected *B. glabrata* snails provided by the NIAID Schistosomiasis Resource Centre of the Biomedical Research Institute (Rockville, MD) through NIH-NIAID Contract HHSN272201700014I and distributed through BEI Resources. Eggs were isolated under sterile conditions from the livers of infected C57BL/6 mice prior to cryopreservation. SEA was prepared by homogenization and ultracentrifugation of *S. mansoni* eggs and concentrated by vacuum dialysis to 1 mg/ml in DPBS (Life Technologies). SEA was fluorescently labeled using an AF660 Microscale Antibody Labelling Kit (Life Technologies) following the manufacturer’s instructions.

### *Heligmosomoides polygyrus* Infection

The *H. polygyrus* life cycle was maintained as previously described (35). For experimental infections, C57BL/6 mice were infected with 200 L3 larvae by oral gavage at six weeks of age. 17 days after infection MLNs and intestines were collected. Adult worm burden was quantified by mounting opened intestines inside a 50 ml falcon filled with PBS. After a 3 h incubation at 37°C, worms were collected from the bottom of the tube and counted under a microscope. HES was collected from adult parasites that were maintained for 21 days in serum-free tissue culture medium and concentrated by vacuum dialysis to 1 mg/ml in DPBS (Life Technologies). HES was fluorescently labeled using an AF660 Microscale Antibody Labelling Kit (Life Technologies) following the manufacturer’s instructions.

### Lymph Node Harvest and Cell Isolation

Individual LNs were identified and collected as described throughout the manuscript and depending on the experiment either all, pooled, or individual lymph nodes were collected. For restimulation cultures 1 × 10^6^ MLN cells were cultured in X-vivo 15 media (Lonza) supplemented with 1% L-glutamine (Invitrogen), 0.1% 2-mercaptoethanol (Sigma-Aldrich), and 7.5 µg/ml SEA or HES in round bottom 96-well plates (Corning) at 37°C and 5% CO2. Supernatants were collected after three days and cytokines detected using the IL-4, IL-5, IL-13, and IFN-*γ* “ready-set-go” ELISA kits (eBioscience) or paired antibodies (Biolegend).

For dendritic cell isolation, lymph was washed in FACS buffer or MLNs were digested with RPMI 1640 (Life Technologies) supplemented with 8 U/ml Liberase and 10 mg/ml DNase (both Sigma-Aldrich) for 45 min at 37°C in a shaking incubator and single-cell suspensions were prepared using a 40 µm cell strainer (Greiner Bio One). For intracellular staining of cytokines, lymph nodes were passed through a 40 µm cell strainer (Greiner Bio One) to create a single cell suspension. 2 × 10^6^ cells were incubated in RPMI 1640 (Life Technologies) supplemented with 2.5 ng/ml PMA (Sigma- Aldrich), 1 mg/ml ionomycin (Invitrogen), 0.5% GolgiStop (BD Bioscience) and 10% FCS for 4 h at 37°C, after which cell surface markers were stained. Cells were fixed and permeabilized using the eBioscience Foxp3/Transcription Factor Staining Buffer Set (eBioscience), and intracellular staining was performed following the manufacturer’s instructions.

### Antibodies for Flow Cytometric Analysis

The following combination of fluorescently labeled primary antibodies against cell surface markers and intracellular cytokines were used: anti-CD3 (17A2), anti-CD4 (clones GK1.5 and RM4-5), anti-CD8a (53–6.7), anti-CD44 (IM7), anti-CD45R/B220 (RA3-6B2), anti-CD11c (N418), anti-CD103 (M290), anti-Ly6C (HK1.4), anti-I-A/I-E (M5/114.15.2), anti-IL17 (TC11-18H10.1), and anti-IL4 (11B11) from Biolegend, and anti-IFN-*γ* (XMG1.2) and anti-IL-13 (eBio13A) from eBioscience. Cells were analyzed using a LSRII flow cytometer running FACSDiva Software (BD Bioscience) and analyzed using FlowJo Software (Tree Star). Fixable Viability Dye eFluor780 (eBioscience), DAPI or 7-AAD Viability Staining Solution (Biolegend) were used according to the manufacturer’s instructions to exclude dead cells from analysis.

### Statistical Analysis

Experimental group sizes ranging from three to five animals were chosen to ensure that a twofold difference between means could be detected with a power of at least 80%. Prism 8 Software (GraphPad) was used to calculate the SEM and the statistical significance using an ordinary one-way ANOVA with Holm–Sidak’s multiple comparisons test, two-way ANOVA with Dunnett’s multiple comparisons test or unpaired t-tests as indicated in the figure legends. Statistical significance was defined as p ≤ 0.05.

## Results

### Increased IFN-*γ* and Decreased Th2 Responses Are Induced When *S. mansoni* Eggs Are Injected Into the Intestinal Subserosa Compared to the Footpad

**Figure 1:**
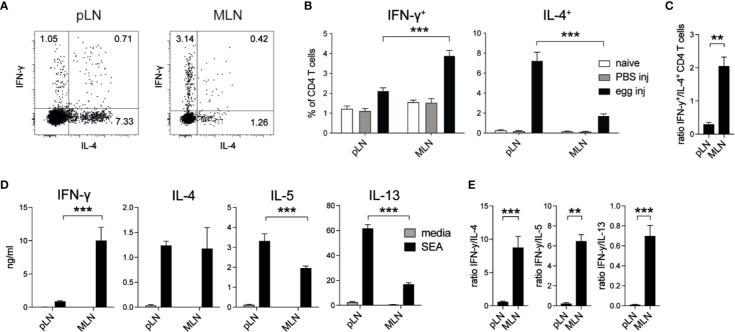
*Schistosoma mansoni* eggs induce increased IFN-*γ* and decreased Th2 responses when injected into the intestine compared to the footpad. **(A, B)** Lymph node IFN-*γ*^+^ and IL-4^+^ CD4^+^ T cell responses in naïve mice or 5 days after the injection of 2,500 *S. mansoni* eggs or PBS in the footpad or intestinal subserosa (n = 5 mice per group, combined data from three independent experiments; mean ± SEM; unpaired t-tests compare LN responses within each experimental group; ***p ≤ 0.001). **(C)** Ratio of IFN-*γ*^+^ and IL-4^+^ CD4^+^ T cell responses after *S. mansoni* egg injection from data shown in **(B)** (mean ± SEM; unpaired t-test compares LN responses; **p ≤ 0.01). **(D)** From the same experiments, lymph node cells were restimulated with soluble egg antigen (SEA) or media for 3 days *in vitro* and Th1 and Th2 cytokines were measured by ELISA (mean ± SEM; unpaired t-tests compare LN cytokine responses within each treatment group; ***p ≤ 0.001). **(E)** Ratio of IFN-*γ* and Th2 cytokine levels after SEA restimulation from data shown in **(D)** (mean ± SEM; unpaired t-test; **p ≤ 0.01, ***p ≤ 0.001).

In previous studies (18, 24, 25), different levels of Th1 and Th2 responses had been observed when *S. mansoni* eggs were injected into different locations, such as the tail vein, the footpad or the intestine. To formally compare whether the injection of *S. mansoni* eggs into different tissues induced different levels of type 1 and type 2 immune responses, we injected 2,500 *S. mansoni* eggs into the footpad or the intestinal subserosa of C57BL/6 mice and collected the draining lymph nodes 5 days after immunization. In line with previous reports, these immunizations resulted in robust Th1 and Th2 responses but revealed differences in the quality of the response when compared to each other. In the popliteal lymph nodes (pLNs) approximately 2% of PMA/ionomycin stimulated CD4^+^ T cells produced IFN-*γ* after egg injection into the footpad, whereas up to 4% of CD4^+^ T cells in the mesenteric lymph nodes (MLNs) were IFN-*γ*^+^ after subserosal egg injection (**Figures 1A, B**; **Supplementary Figure 1A**). Conversely, in the same experiment IL-4^+^ T cells were four times more abundant in the pLNs than in the MLNs, resulting in a much higher ratio of IFN-*γ*^+^ T cells in the MLNs (**Figure 1 C**). As the subserosal injection required laparotomy surgery, we also assessed IFN-*γ* and IL-4 responses under mock surgery conditions and confirmed that the injection itself did not induce IFN-*γ* or IL-4 T cell responses, with no significant levels of IFN-*γ*^+^ or IL-4^+^ T cells detected in naïve or PBS injected mice (**Figure 1B**).

To assess antigen-specific cytokine responses in these experiments, we restimulated pLN or MLN cells with soluble egg antigen (SEA) for three days and measured cytokine production by ELISA. Similar to our observations for PMA/ionomycin stimulated CD4^+^ T cells, high levels of IFN-*γ* were produced by MLN cells after subserosal egg injection, whereas pLN cells secreted higher levels of IL-5 and IL-13 after egg injection into the footpad (**Figure 1D**). Despite similar levels of IL-4 production in the pLN and MLN, the ratio of secreted IFN-*γ* compared to each of the Th2 cytokines was always greater in the MLN (**Figure 1E**).

Thus, increased antigen-specific IFN-*γ* and reduced Th2 responses were observed in the draining LNs when *S. mansoni* eggs were injected into the intestine compared to the footpad, indicating that tissue-specific signals can influence the balance of type 1 and type 2 immunity after experimental immunization.

### Th1 and Th2 Responses Develop in Individual Intestinal Lymph Nodes After Live *S. mansoni* Infection or Experimental Egg Immunization

To assess if the site of *S. mansoni* egg deposition during live infection also affected type 1 and type 2 immune responses in the intestinal draining LNs, we infected C57BL/6 mice with 180 *S. mansoni* cercariae and analyzed the MLNs after 7 weeks, which coincides with the onset of egg production (15).

**Figure 2:**
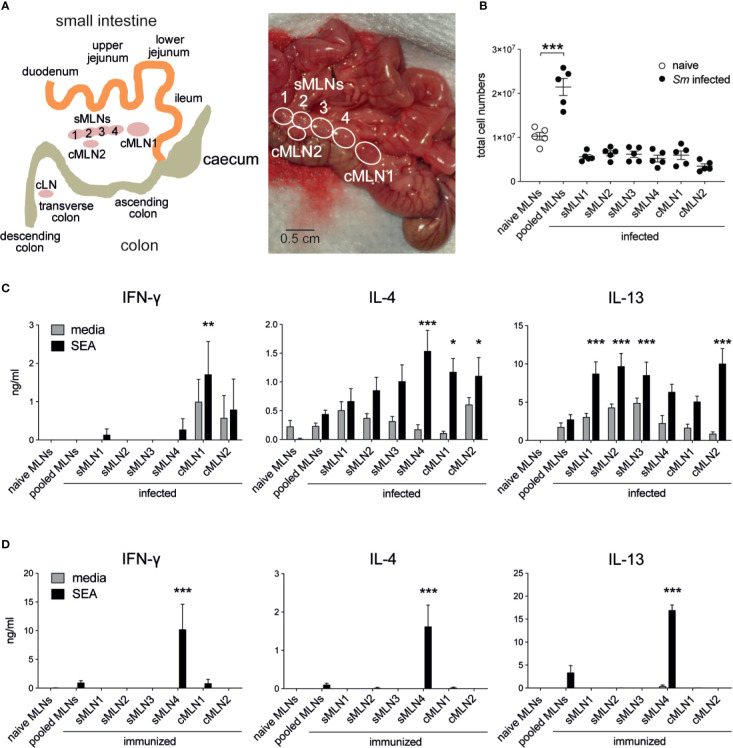
Th1 and Th2 responses are detected in individual mesenteric lymph nodes after *Schistosoma mansoni* infection and egg immunization. **(A)** Schematic and photograph of the murine intestine illustrate the different segments and indicate the position and nomenclature of the individual draining lymph nodes. **(B)** Total cell counts of pooled or individual mesenteric lymph nodes (MLNs) seven weeks after *S. mansoni* (*Sm*) infection (n = 5 mice per group, representative of two independent experiments; mean ± SEM; unpaired t-tests compare cell numbers between naïve and infected groups; **p ≤ 0.01). **(C)** From the same experiments, pooled or individual lymph node cells were restimulated with SEA or media for 3 days *in vitro* and Th1 and Th2 cytokines were measured by ELISA (mean ± SEM; two-way ANOVA followed by Dunnett’s multiple comparisons test compare samples to pooled infected MLNs within each restimulation group; *p ≤ 0.05, **p ≤ 0.01, ***p ≤ 0.001). **(D)** 1,000 *S. mansoni* eggs were injected into the ileal subserosa and pooled or individual lymph nodes were collected after 5 days. Cells were restimulated with SEA or media for 3 days *in vitro* and Th1 and Th2 cytokines were measured by ELISA (n = 3 mice per group, combined data from three independent experiments; mean ± SEM; two-way ANOVA followed by Dunnett’s multiple comparisons test compare samples to pooled infected MLNs within each restimulation group; ***p ≤ 0.001).

Although egg granuloma formation has been observed both in the small and large intestines (36, 37), it remains unclear which regions of the intestine are most affected and if different levels of immune responses are initiated. To monitor antigen-specific T cell immunity in the segment-specific draining LNs we identified the individual mesenteric LNs, according to previous reports (25, 30, 38–40). We termed the separate larger LN closest to the caecum ‘cMLN1’ (colon draining MLN 1), the adjacent string of four similar sized LNs draining the small intestine ‘small intestine draining MLNs 1–4’ (sMLN1–4), and the smaller lymph nodes draining the lower colon and the caudal LN ‘cMLN2’ and ‘cLN’, respectively (**Figure 2A**).

To assess location-dependent antigen-specific immune responses during live infection, LNs were individually collected and cultured for three days with media or SEA. As expected, MLN cellularity increased after infection, due to activation of the immune system by eggs passing through or becoming lodged in the intestinal tissue (**Figure 2B**), and the cytokine profile of cultured LN cells revealed strong immune activation. Significant levels of IFN-*γ*, IL-4 and IL-13 were observed in the absence of antigen and increased after SEA restimulation (**Figure 2C**). In contrast to previous studies (24, 41), levels of IFN-*γ* were low in our study, and IFN-*γ* was not detectable in pooled MLNs. However, IL-4 and IL-13 were detected at similar levels to those previously described (24, 41). Peak IL-4 responses were observed in sMLN4, cMLN1, and cMLN2, whereas peak IL-13 responses were observed in sMLN1, sMLN2, sMLN3, and cMLN2. IFN-*γ* could only be detected in the colon draining LNs cMLN1 and cMLN2, suggesting a bias towards Th1 responses in the large intestine.

It is difficult to determine whether the T cell responses observed in the individual LNs during live infection resulted from T cells primed within that same individual lymph node or had recirculated from other sites. To develop a more controlled model, we delivered *S. mansoni* eggs directly into the ileal subserosa and assessed T cell responses 5 days later. A single injection of 1,000 eggs into the ileum was sufficient to drive a robust antigen-specific T cell response in the draining LNs (**Figure 2D**), which were only detected in one individual LN (sMLN4), indicating that T cell priming occurs in individual LNs that drain the injection site.

In summary, both live *S. mansoni* infection and *S. mansoni* egg injection elicited robust antigen-specific immune responses in the MLNs but showed clear differences depending on which individual LNs were affected. All individual LNs draining the small and large intestines mounted similar levels of Th2 responses, while Th1 responses were restricted to the LNs draining the large intestine after live infection, whereas *de novo* primed T cells only responded in an individual LN after subserosal egg immunization.

### *H. polygyrus* Antigens Induce Increased IFN-*γ* and Decreased Th2 Responses When Injected Into the Intestinal Subserosa Compared to the Footpad

To test whether tissue-specific differences in the induction of type 1 and type 2 immune responses would also affect other helminth antigens, we assessed immune responses against *Heligmosomoides polygyrus*, a gut dwelling nematode.

**Figure 3:**
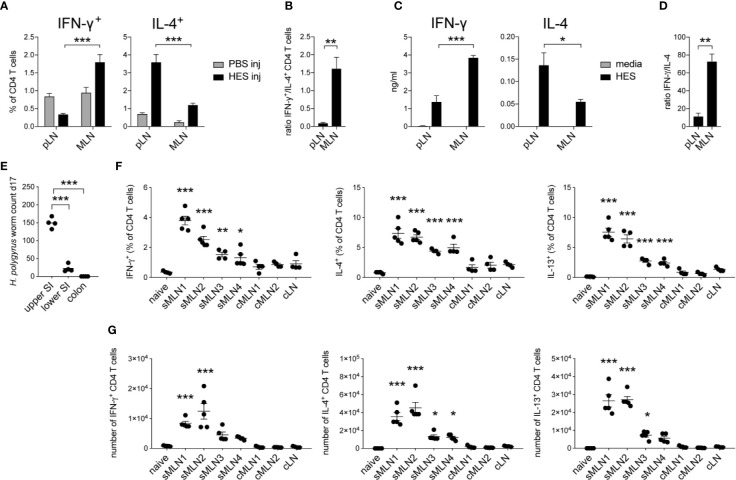
*Heligmosomoides polygyrus* antigens induce stronger Th1 and reduced Th2 responses in the intestinal draining lymph nodes compared to the footpad. **(A)** 20 µg of *H. polygyrus* ES antigen (HES) was injected in the footpad or ileal subserosa. IFN-*γ*^+^ and IL-4^+^ CD4^+^ T cell responses were analyzed 5 days after injection in the popliteal (pLN) or mesenteric (MLN) lymph nodes (n = 5 mice per group, combined data from two independent experiments; mean ± SEM; unpaired t-tests compare LN responses within each experimental group; ***p ≤ 0.001). **(B)** Ratio of IFN-*γ*^+^ and IL-4^+^ CD4^+^ T cell responses after HES injection as shown in **(A)** (mean ± SEM; unpaired t-test compares LN responses; **p ≤ 0.01). **(C)** From the same experiments, LN cells were restimulated with HES or media for 3 days *in vitro* and Th1 and Th2 cytokines were measured by ELISA (mean ± SEM; unpaired t-tests compare LN responses within each experimental group; *p ≤ 0.05, ***p ≤ 0.001). **(D)** Ratio of IFN-*γ* and IL-4 cytokine levels after HES restimulation as shown in **(C)** (mean ± SEM; unpaired t-test compares LN responses; **p ≤ 0.01, ***p ≤ 0.001). **(E)** Worm counts from the upper half of the small intestine (upper SI), lower half of the small intestine (lower SI) and the colon 17 days after infection with 200 L3 *H. polygyrus* larvae (n = 4 mice, representative of two independent experiments; mean ± SEM; unpaired t-tests compare worm counts to upper SI numbers; ***p ≤ 0.001). **(F, G)** Mice were infected with 200 L3 *H. polygyrus* larvae by oral gavage and individual MLNs were collected after 17 days. Frequency and number of IFN-*γ*, IL-4, and IL-13 producing CD4^+^ T cells in the individual MLNs are shown (n = 5 mice per group, representative of two independent experiments; mean ± SEM; ordinary one-way ANOVA followed by Holm–Sidak’s multiple comparisons test compare LN responses to naïve controls; *p ≤ 0.05, **p ≤ 0.01, ***p ≤ 0.001).

Soluble excretory/secretory antigens from *H. polygyrus* (HES) were injected into the footpad or the intestinal subserosa of C57BL/6 mice and IFN-*γ*^+^ and IL-4^+^ CD4^+^ T cells or cytokine production after HES restimulation were assessed in the respective draining LNs after 5 days. Similar to our observations after *S. mansoni* egg injection, more IFN-*γ*^+^ CD4^+^ T cells were observed in the MLNs after HES injection into the ileal subserosa compared to injections into the footpad, whereas IL-4^+^ CD4^+^ T cells were more abundant in the pLNs after footpad injection (**Figure 3A**, **Supplementary Figure 2A**). This increased ratio of IFN-*γ*^+^ CD4^+^ T cells after subserosal injection (**Figure 3B**) was also observed after *in vitro* restimulation of LN cells with HES and showed increased levels of antigen-specific IFN-*γ* and decreased levels of IL-4 after subserosal injection, compared to injections into the footpad (**Figures 3C, D****)**. The observation that greater antigen-specific IFN-*γ* and reduced Th2 responses were observed in the MLNs against helminth products from both *S. mansoni* and *H. polygyrus* suggests that tissue-specific mechanisms can influence the balance of type 1 and type 2 immune responses against multiple helminth antigens.

To assess CD4^+^ T cell responses in individual intestinal draining MLNs after live *H. polygyrus* infection, C57BL/6 mice were infected with 200 L3 *H. polygyrus* larvae by oral gavage and the individual MLNs were collected after 17 days. Similar to previous reports (42–44), the majority of adult worms were detected in the upper small intestine (SI), with few worms present in the lower SI and no worms present in the colon (**Figure 3E**). Correspondingly, we observed a significant increase in total cell numbers, CD4^+^ T cells and IFN-*γ*^+^, IL-4^+^ and IL-13^+^ T cells in the individual MLNs draining the proximal intestine, with responses declining in the MLNs that drained the lower areas of the intestine (**Figures 3F, G**, **Supplementary Figures 2B, C****)**.

### Individual Mesenteric Lymph Nodes Drain Specific Intestinal Segments After Subserosal Dye Injection

To identify what exact segments of the intestine were drained by these individual MLNs we set out to map the lymphatic drainage within the intestine *in vivo*. The luminal injection of dyes (30, 38, 39) or gavaging of olive oil (45) **(****Supplementary Figure 1B**) has previously allowed researchers to map intestinal regions to individual LNs. Yet, due to the diffusion and uptake characteristics of the different dyes that were used in these studies, a consistent assessment of all regions of the intestine is still missing.

**Figure 4:**
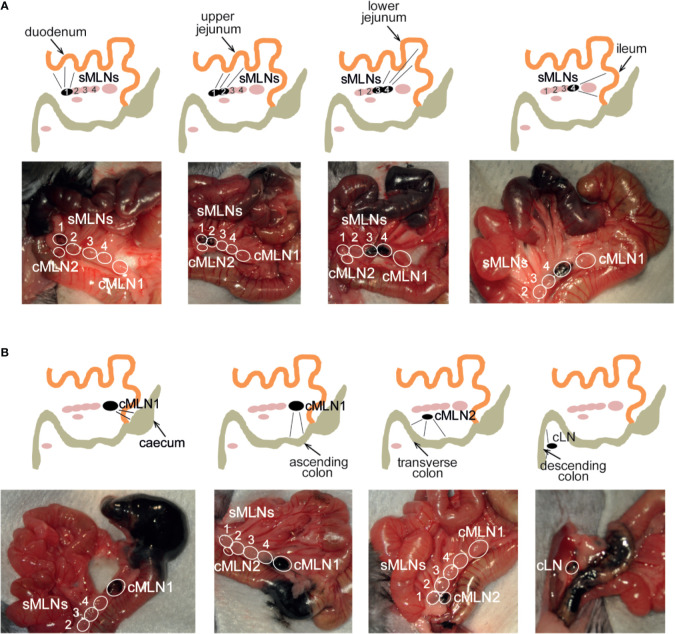
Subserosal dye injection along the murine intestine identifies individual segment draining lymph nodes. Anesthetized animals were injected with 10 µl of black calligraphy ink into different segments of the small **(A)** or large intestine **(B)**. Photographs were taken 1 min after dye injection. Schematics indicate injection site, labeled lymphatics and individual draining lymph nodes as seen in each photograph.

To identify a dye that would result in fast uptake and labeling of the lymphatics, while displaying limited diffusion characteristics, we trialled the injection of different dyes into the intestinal subserosa of anesthetized animals and monitored the lymphatic vessels and LNs for uptake of the dye. Similar to previous studies (38, 39), 2% Chicago Blue or 2% Evans Blue rapidly diffused along the intestine, the lymphatics and the MLNs, making it difficult to detect segment-specific draining LNs, while a suspension of graphite particles or black India ink resulted in a very localized deposition, but no uptake by the lymphatics. In contrast, the injection of black calligraphy ink resulted in localized coloration of the injected segment and lymphatic drainage to individual MLNs within seconds (**Figure 4**, **Supplementary Video 1**). In line with previous findings (30, 38–40), subserosal dye injection into the duodenum labeled sMLN1, while injections into the upper jejunum, lower jejunum or ileum labeled sMLN1 and sMLN2, sMLN3 and sMLN4 or sMLN4 respectively (**Figure 4A**). Subserosal dye injections into different segments of the large intestine demonstrated that injections into the caecum or ascending colon labeled cMLN1, while the transverse or descending colon was drained by cMLN2 or the cLN, respectively (**Figure 4B**). By subserosal injection of a dye that had fast lymphatic uptake and limited diffusion characteristics, we were thus able to map the lymphatic drainage of all segments of the intestine and define the individual segment-specific draining lymph nodes.

### Individual Mesenteric Lymph Nodes Mount Distinct Th1 and Th2 Responses Against Subserosally Injected *S. mansoni* Eggs

As we had observed distinct LN-specific immune responses after live *S. mansoni* infection, we investigated whether *S. mansoni* eggs would induce similar LN-specific responses after controlled egg delivery into different sites of the intestine. To precisely control the dose, location, and timing of helminth antigen delivery, 1,000 *S. mansoni* eggs were injected subserosally into each segment of the small and large intestines. After 5 days individual MLNs were collected, LN cells were restimulated with SEA for 3 days *in vitro* and antigen-specific cytokine responses were measured by ELISA.

**Figure 5:**
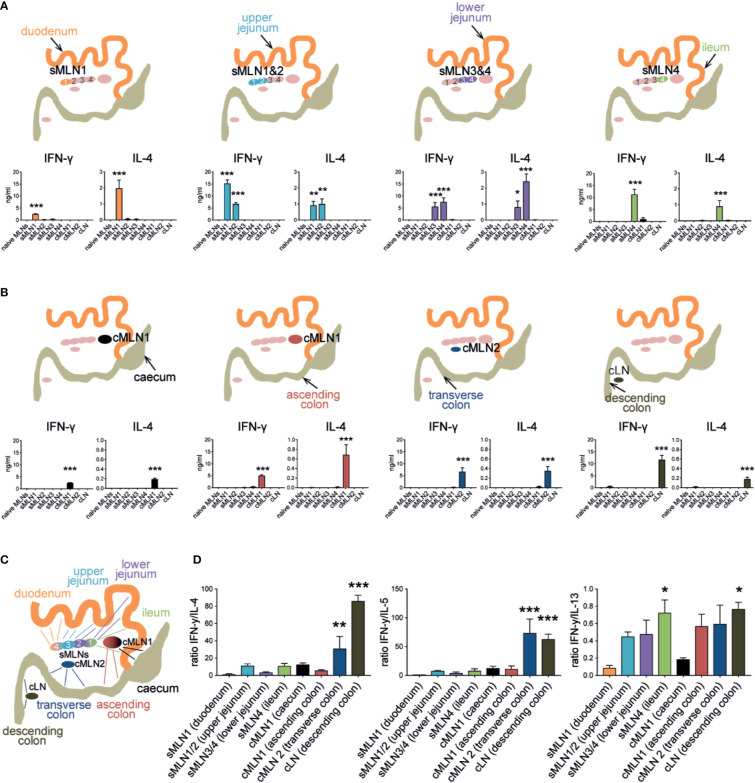
Subserosal injection of *Schistosoma mansoni* eggs induces segment-specific Th1 and Th2 responses in the individual MLNs. **(A)** 1,000 *S. mansoni* eggs were injected into different segments of the small intestine. Individual MLNs were collected five days after injection; LN cells were restimulated with SEA *in vitro* and IFN-*γ*, and IL-4 were measured by ELISA. Schematics indicate injection site and responding LNs (n = 3 mice per group, combined data from three independent experiments; mean ± SEM; ordinary one-way ANOVA followed by Holm–Sidak’s multiple comparisons test compare LN responses to naïve controls; *p ≤ 0.05, **p ≤ 0.01, ***p ≤ 0.001). **(B)** Similar to **(A)**, 1,000 *Schistosoma mansoni* eggs were injected into the different segments of the large intestine. After 5 days LNs were collected, restimulated *in vitro*, and assessed for antigen-specific cytokines by ELISA (n = 3 mice per group, combined data from three independent experiments; mean ± SEM; ordinary one-way ANOVA followed by Holm–Sidak’s multiple comparisons test compare LN responses to naïve controls; ***p ≤ 0.001). **(C)** Schematic of the murine intestine indicating the draining pattern of the different intestinal segments to their individual MLNs. **(D)** Segment-specific ratio of IFN-*γ* and Th2 cytokine levels after SEA restimulation from experiments described in **(A, B)** and **Supplementary Figure 3** (mean ± SEM; ordinary one-way ANOVA followed by Holm–Sidak’s multiple comparisons test compare samples to sMLN1 ratios; *p ≤ 0.05, **p ≤ 0.01, ***p ≤ 0.001).

We detected IFN-*γ*, IL-4, IL-5 and IL-13 responses in the draining LNs of all immunized groups, which were localized to the same segment-specific LNs as identified by our ink injection experiments. We observed that the cytokine levels generated from the small intestine-draining MLNs ranged from 2 to 15 ng/ml for IFN-*γ*, 1 to 3 ng/ml for IL-4, 0.5 to 2 ng/ml for IL-5, and 5 to 35 ng/ml for IL-13 after egg injection into the different segments of the small intestine. Of note, injection of the duodenum resulted in a low IFN-*γ* response (2 ng/ml) compared to the other small intestinal sites, whereas IL-4 and IL-5 responses were comparable, and IL-13 responses decreased along the small intestine (**Figure 5A**, **Supplementary Figure 3A**).

Injection of eggs into specific regions of the large intestine resulted in lower IL-4, IL-5, and IL-13 responses in the LNs compared to the small intestine, which ranged from 0.2 to 0.8 ng/ml for IL-4, 0.1 to 0.5 ng/ml for IL-5 and 10 to 20 ng/ml for IL-13, whereas IFN-*γ* responses were in a similar range (2–12 ng/ml). After caecal injection, cMLN1 exhibited low responses of both IFN-*γ* and IL-4, compared to the other large intestinal injections, whereas the cLN displayed high levels of IFN-*γ* accompanied by lower levels of IL-4 and IL-5 after egg injection into the descending colon (**Figure 5B**, **Supplementary Figure 3B**). Both, small and large intestinal immune responses were localized to the same segment-specific LNs that we had identified in our ink injection experiments (summarized in **Figure 5C**), clearly demonstrating that site-specific immune responses were induced, which varied between the responding LNs.

When we analyzed the ratio of IFN-*γ* to the different Th2 cytokines in the individual MLNs after segment-specific subserosal *S. mansoni* egg injection, we observed patterns that indicated gradual changes in the ratio of type 1 and type 2 immune profiles along the intestine (**Figure 5D**). A low ratio of IFN-*γ* to Th2 responses was observed in the LNs draining the proximal intestine, with the lowest ratio of IFN-*γ* to all Th2 cytokines observed in sMLN1 after duodenal injection. A pronounced increase in the ratio of IFN-*γ* to IL-4 and IL-5 was observed in cMLN2 and cLN after subserosal egg injections in the transverse and descending colon due to low levels of Th2 cytokines being detected in these LNs (**Figure 5B**, **Supplementary Figure 3B**), demonstrating a distinct and increasing proportion of IFN-*γ* responses in the distinct LNs draining the length of the intestine.

The ratio of IFN-*γ* to IL-4 and IL-5 responses in the individual draining LNs correlated well with each other, while IL-13 responses followed a different pattern, similar to our observations during live *S. mansoni* infection (**Figure 2C**). IL-13 levels gradually decreased throughout the LNs draining the small intestine from 30 ng/ml in sMLN1 to 15 ng/ml in sMLN4 and stayed within a range of 15–20 ng/ml in the large intestinal draining LNs. The resulting IFN-*γ* to IL-13 ratio thus increased gradually within the LNs draining the length of the small intestine, decreased in cMLN1 after caecal injection (due to low IFN-*γ* levels) and returned to a higher ratio for the remaining large intestinal LNs.

While our model does only approximate the individual LN immune responses, it suggests that different levels of antigen-specific type 1 and type 2 cytokines are produced in individual intestinal LNs after controlled immunization of different intestinal segments with *S. mansoni* eggs.

### Different Subsets of Intestinal Dendritic Cells Transport and Present Soluble Helminth Antigens in the MLNs

**Figure 6:**
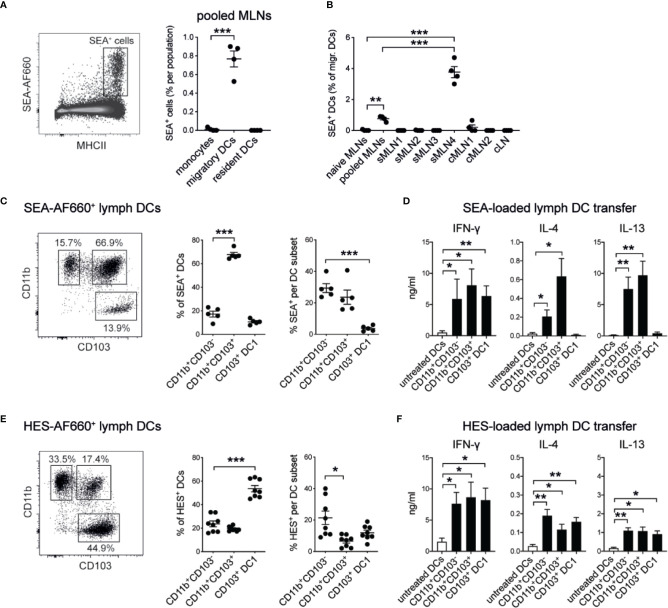
Soluble helminth antigens are transported to the MLN by distinct subsets of intestinal dendritic cells that prime Th1 and Th2 responses. 15 µg of AF660-labeled antigens were injected into the intestinal serosa and antigen-positive cells were assessed 24 h after injection. **(A)** Representative dot plot of viable single MLN cells and frequency of SEA-AF660^+^ cells in pooled MLNs 24 h after ileal injection (n = 4 mice per group, representative of two independent experiments; mean ± SEM; unpaired t-tests compare cell frequencies to monocytes; ***p ≤ 0.001). **(B)** Frequency of SEA-AF660^+^ dendritic cells in pooled or individual MLNs 24 h after ileal injection (n = 4 mice per group, representative of two independent experiments; mean ± SEM; ordinary one-way ANOVA followed by Holm–Sidak’s multiple comparisons test compares DC frequencies to naïve controls; unpaired t-test compares pooled MLN to sMLN4 responses; **p ≤ 0.01, ***p ≤ 0.001). **(C)** DC subset distribution (left and middle) and frequency (right) of SEA-AF660^+^ lymph DCs collected over 18 h after ileal injection of SEA-AF660 (n = 2–3 mice per group, combined data from two independent experiments; mean ± SEM; unpaired t-tests compare cell distribution and frequencies to CD11b^+^CD103^−^ DCs; ***p ≤ 0.001). **(D)** Lymph DC subsets were collected from naïve mice over 18 h, sorted into subsets and incubated with or without 1 mg/ml SEA for 18 h. 30,000 cells were then transferred under the MLN capsule of naïve mice. After 5 days LNs were collected, restimulated with SEA *in vitro*, and assessed for antigen-specific cytokines by ELISA (n = 2–3 mice per group, combined data from two independent experiments; mean ± SEM; unpaired t-tests compare cytokine responses from transferred SEA-treated DC subsets to untreated DCs; *p ≤ 0.05, **p ≤ 0.01). **(E)** DC subset distribution (left and middle) and frequency (right) of HES-AF660^+^ lymph DCs collected over 18 h after ileal injection of HES-AF660 (n = 2–3 mice per group, combined data from two independent experiments; mean ± SEM; unpaired t-tests compare cell distribution and frequencies to CD11b^+^CD103^−^ DCs; *p ≤ 0.05, ***p ≤ 0.001). **(F)** Lymph DC subsets were collected from naïve mice over 18 h, sorted into subsets and incubated with or without 1 mg/ml HES for 18 h. 30,000 cells were then transferred into the MLN subcapsule of naïve mice. After 5 days LNs were collected, restimulated with HES *in vitro*, and assessed for antigen-specific cytokines by ELISA (n = 2–3 mice per group, combined data from two independent experiments; mean ± SEM; unpaired t-tests compare cytokine responses from transferred HES-treated DC subsets to untreated DCs; *p ≤ 0.05, **p ≤ 0.01).

Dendritic cells (DCs) play a crucial role in priming CD4^+^ T cell responses against parasite antigens (25, 46–50). To confirm that DCs were also responsible for the uptake of subserosally injected helminth antigens and their delivery to the individual draining MLNs, we injected fluorescently labeled AF660-SEA into the ileum, collected the individual MLNs 24 h after injection, and assessed antigen-positive cells by flow cytometry. As expected, MHCII^hi^ CD11c^+^ migratory DCs were the only antigen-presenting cell population labeled with AF660-SEA in the MLNs (**Figure 6A**, **Supplementary Figure 4A**). Around 1% of DCs were SEA-positive in pooled MLNs, whereas up to 4% SEA^+^ DCs were detected in sMLN4 but no other individual LN (**Figure 6B**). Thus, locally delivered antigens were transported to the individual MLN that we had previously identified, and a dilution of signal occurred when LNs were pooled.

To investigate which specific subsets of migratory DCs were involved in the uptake and active transport of helminth antigens from the intestine to the draining MLNs, we injected fluorescently labeled SEA or HES into the ileum of mesenteric lymphadenectomized (MNLx) mice, performed thoracic duct cannulations and collected migrating intestinal dendritic cells over the course of 18 h. We observed that SEA was mainly associated with CD11b-positive DCs, as previously reported (25) and that CD11b^+^CD103^+^ DCs were the most frequent SEA^+^ migratory DC population (**Figure 6C**). To investigate if these DC subsets were also sufficient to prime antigen-specific immune responses, we transferred antigen-loaded DCs into naïve MLNs using microsurgical techniques we had previously developed (25, 34). We collected lymph DC subsets from naïve MLNx mice and loaded them with SEA *in vitro* for 18 h to ensure a controlled uptake of the antigen. After washing off excess antigen, 30,000 cells of each DC subset were transferred under the MLN capsule of naïve mice to assess if these subsets were sufficient to drive antigen-specific immune responses. After 5 days the injected MLNs were collected, cells were cultured with SEA for 3 days *in vitro*, and antigen-specific cytokine responses were assessed by ELISA. Similar to our previous findings (25) and in accordance with the observation that CD11b-positive DCs were responsible for transporting SEA *in vivo*, we found that both CD11b^+^CD103^−^ and CD11b^+^CD103^+^ DC2 subsets were able to prime IFN-*γ* and Th2 cytokine producing T cell responses, when antigen-loaded DCs were transferred under the MLN capsule (**Figure 6D**). CD103 single-positive DC1s were also able to induce antigen-specific immune responses in this system but did only induce IFN-*γ* responses with no IL-4 or IL-13 being detected.

In contrast, HES was mainly transported by CD11b^+^CD103^−^ DC2s and CD103^+^ single-positive DC1s in lymph, while CD11b^+^CD103^+^ double-positive DCs were the least frequent HES^+^ DCs (**Figure 6E**). Similar, to our observation of SEA-loaded DCs, HES-specific IFN-*γ* responses were induced by all three migratory DC subsets, when HES-loaded DC subsets were transferred under the MLN capsule (**Figure 6F**). In contrast to SEA-loaded DCs, all HES-loaded DC subsets induced similar levels of IL-4 and low levels of IL-13 responses, indicating that HES is transported and presented by all subsets of intestinal migratory DCs to drive Th2 responses that show a different cytokine profile and ratio of immune responses than those observed with SEA (**Supplementary Figure 4B**).

Thus, location-specific variation of the immune response, as well as antigen-dependent uptake and presentation by different subsets of intestinal dendritic cells, likely shape the resulting immune responses to helminth antigens, resulting in a complex regulation of local type 1 and type 2 immunity.

## Discussion

In this study we report that the delivery of helminth antigens from *S. mansoni* and *H. polygyrus* into the intestine promotes higher Th1 and lower Th2 responses in the draining LNs compared to delivery into the footpad. In a more detailed analysis of intestinal immune responses initiated during live infection or by experimentally delivering *S. mansoni* eggs into different sites within the intestine itself, we furthermore show that distinct levels of type 1 and type 2 immune responses develop in the individual draining MLNs, with higher ratios of IFN-**γ** responses associated with infection/immunization of the large compared to the small intestine. Soluble helminth antigens are hereby transported by lymph migrating DCs, and different DC subsets transport and prime antigen-specific immune responses in an antigen-dependent manner in the draining MLNs.

Our observation that the controlled delivery of the same antigen into different sites of the intestine induces distinct Th1 and Th2 responses shares similarities with a previous study by Esterházy et al. (30), which demonstrates that the preferential development of Th17 or T regulatory cell responses is determined by location-specific signals within the intestine that include distinct stromal and dendritic cell signatures.

While LN stromal cells have been shown to influence T cells homing (51), support T regulatory cell induction (51), and limit or support inflammatory T cells during immunization or infection (52, 53), little is known if LN stromal cells also play a role in type 2 immunity against helminths. While helminth infection has been shown to promote stromal cell remodeling and *de novo* B cell follicle formation to promote total and helminth-specific antibody production (54), with IL-4 being critical to promote stromal cell expansion (55), it remains unknown if LN stromal cells can also directly affect Th2 development.

The involvement of DCs in the induction of type 2 immune responses against helminths is in turn better understood (47). CD11c^+^ DCs are necessary for the induction of type 2 immune responses against *S. mansoni* and SEA *in vivo* (46) and IRF4-dependent migratory DC2s are required for effective type 2 immunity in the intestine (25, 48), lung (56), and skin (48, 57). Further DC subsets that express CD301b or are dependent on KLF4 are required to drive type 2 immune responses in the skin (49, 58, 59) but have not been described in other tissues (47). Our comparison of type 2 immune responses after intestinal or footpad immunization of SEA (**Figure 1**) or HES (**Figure 3**) demonstrates that IL-4^+^ cells are more frequent and Th2-associated cytokine levels are higher after restimulation in the pLN compared to the MLN, indicating that the skin represents a Th2-promoting environment, which could be influenced by skin-specific subsets of DCs.

While the proportion of DC2 subsets within the intestine changes from a dominant CD11b^+^CD103^+^ phenotype in the small intestine to a CD11b^+^CD103^−^ phenotype in the colon (60, 61), both populations are sufficient to drive type 2 immune responses against *S. mansoni* eggs (25) and can drive SEA-specific immune responses when transferred *in vivo* (**Figure 6**). Our observation that SEA and HES display distinct uptake characteristics by migratory intestinal DC subsets and promote different levels of IL-4 and IL-13 immune responses when transferred *in vivo* (**Figure 6**) suggests that type 2 immune responses are mounted in a highly antigen-dependent manner, which could elicit distinct location dependent immune responses within the intestine.

We show that in contrast to SEA and most reported Th2-inducing antigens (47), which are presented by IRF4^+^ DC2s, intestinal BATF3-dependent CD103^+^ single-positive DCs (62) can also take up fluorescently labeled HES and are sufficient to induce HES-specific immune responses when transferred *in vivo* (**Figure 6**). While the direct involvement of CD103 single-positive DC1s in priming Th2 responses in this system requires further investigation, BATF3-dependent migratory DCs are known to suppress helminth-driven type 2 immunity in the intestine through the expression of IL-12 (63). As CD103^+^ single-positive DCs are most prominent in the distal intestine (60, 61), it is possible that this subset could directly or indirectly contribute to the increased IFN-*γ* responses (**Figure 2C**) and IFN-*γ* to Th2 ratio (**Figure 5**) that we observed in the LNs draining the large intestine.

Apart from cellular differences that exist within the LNs, several studies have also shown that antigen-specific immune responses in the intestine and MLNs can be affected by the intestinal microbiota. A direct link between anti-helminth immunity and the microbiota was observed in antibiotic-treated *S. mansoni* infected mice that develop smaller granulomas and produce lower levels of IFN-*γ* in the intestine (41). Similar observations have been made in *Myd88*^−/−^ mice, which cannot respond to TLR signals, and displayed reduced IFN-*γ* but intact Th2 responses after *S. mansoni* infection (64). As *S. mansoni* eggs are themselves weak inducers of TLR responses compared to bacterial compounds such as LPS (64–66), it is likely that these changes are the result of defective microbiota signaling. Bacterial compounds have also been shown to directly act on DCs and limit their potential to drive type 2 immune responses (67, 68) and could also act through innate immune cells that in turn alter their production of Th2-stimulting alarmins. As increasing microbial burden and diversity have been reported along the length of the intestine (60, 69), it is conceivable that they could also influence the ratio of Th1 and Th2 responses after *S. mansoni* egg immunization along the intestine. Experiments in germ-free or antibiotic-treated mice would clarify to what extend the microbiota is involved in regulating site-specific anti-helminth immune responses.

Given the co-evolutionary development between helminths and their hosts, several factors likely influence local immune responses and directly or indirectly modulate anti-helminth Th2 immunity to create a favorable immunological environment for the parasite. Our study demonstrates that such site-specific differences exist between the skin and the intestine, and that within the intestine *S. mansoni* infection promotes distinct levels of type 1 and type 2 responses in individual MLNs, which we also observe when a controlled dose of *S. mansoni* eggs is experimentally delivered into distinct segments of the intestine. While we could not determine if these location-dependent differences are influenced by distinct immune cell populations or external stimuli, our observation suggests that Th1 and Th2 responses against helminth antigens are distinctly regulated in different regions of the intestine.

## Data Availability Statement

All datasets presented in this study are included in the article/**Supplementary Material**.

## Ethics Statement

Animal experiments carried out at the University of Glasgow were performed under licenses issued by the UK Home Office (Project License 60/4500) and were approved by the Ethical Review Panel of the University of Glasgow. Animal experiments carried out at the University of Manchester adhered to UK Home Office regulations (project license 70/7815) and were approved by the Ethical Review Panel of the University of Manchester. Animal experiments carried out at the Malaghan Institute of Medical Research adhered to the regulations of the Ministry of Primary Industries New Zealand (licenses 2014R22M, 24432) and were approved by the Animal Welfare Ethical Review Board of the Victoria University of Wellington.

## Author Contributions

JM and SLB performed the experiments. ASM provided reagents and, with SM, helped direct the study. JM conceived the project and together with SM and ASM wrote the manuscript. All authors contributed to the article and approved the submitted version.

## Funding

This work was supported by a Wellcome Trust PhD studentship (099784/Z/12/Z) to JM, by a grant from the Medical Research Council (MR/K021095/1) to SM and and core funding from the Manchester Collaborative Centre for Inflammation Research (MCCIR) to AM.

## Conflict of Interest

The Manchester Collaborative Centre for Inflammation Research (MCCIR) is a joint venture between the University of Manchester and GlaxoSmithKline. SLB and ASM were employed by The Manchester Collaborative Centre for Inflammation Research (MCCIR).

The remaining authors declare that the research was conducted in the absence of any commercial or financial relationships that could be construed as a potential conflict of interest.
