# Supplementary material for: Defined Intestinal Regions Are Drained by Specific Lymph Nodes That Mount Distinct Th1 and Th2 Responses Against Schistosoma mansoni Eggs
Source: Front Immunol. 2020 Oct 23;11:592325. doi: 10.3389/fimmu.2020.592325 (PMC7644866; doi:10.3389/fimmu.2020.592325)
Supplement: Supplementary file 2 [file DataSheet_2.pdf]

A

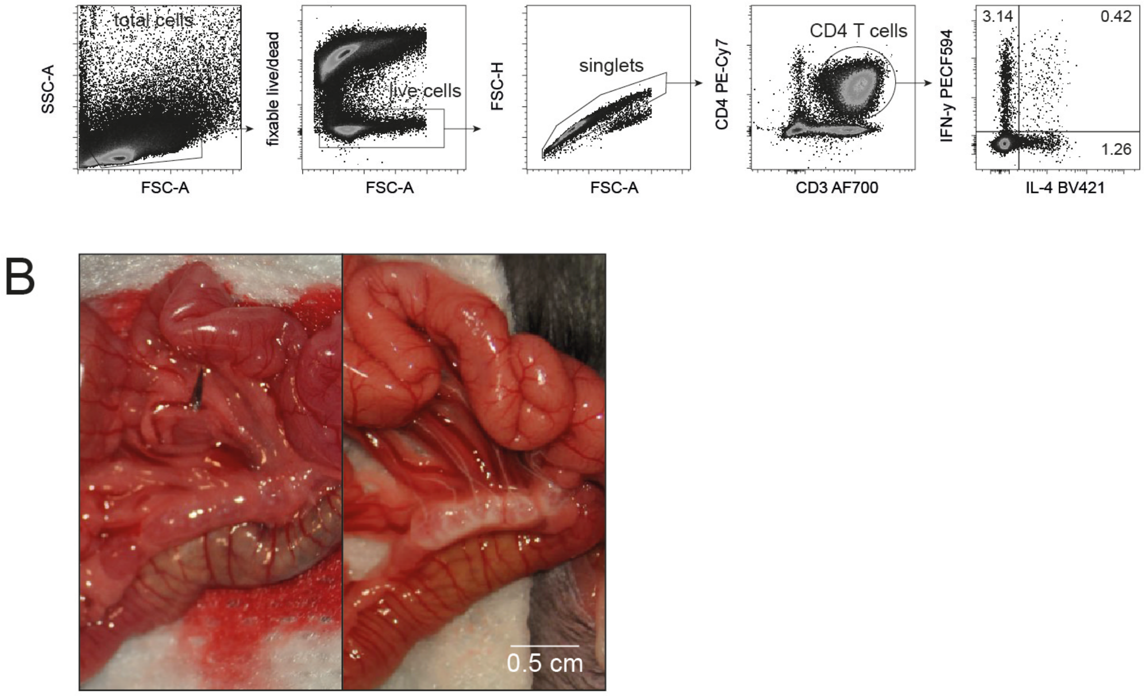

Supplementary Figure 1 (related to Main Figure 1). Gating strategies and intestinal lymphatic drainage visualisation. (**A**) 2,500 *Schistosoma mansoni* eggs or PBS were injected into the footpad or intestinal subserosa. Five days after immunization lymph nodes were collected, stimulated with PMA/ionomycin for four hours and analysed by flow cytometry. IFN-γ and IL-4 producing CD4 T cells were identified by gating on viable single cells that expressed the T cell markers CD3 and CD4. Representative dot plots for the MLN of an immunized mouse are shown. (**B**) To visualise intestinal lymphatic drainage, mice were gavaged with 0.3 ml olive oil. Laparotomy surgery was performed 30 minutes after gavage and photographs of the murine intestine of a naïve (left) and oil gavaged (right) mouse are shown.

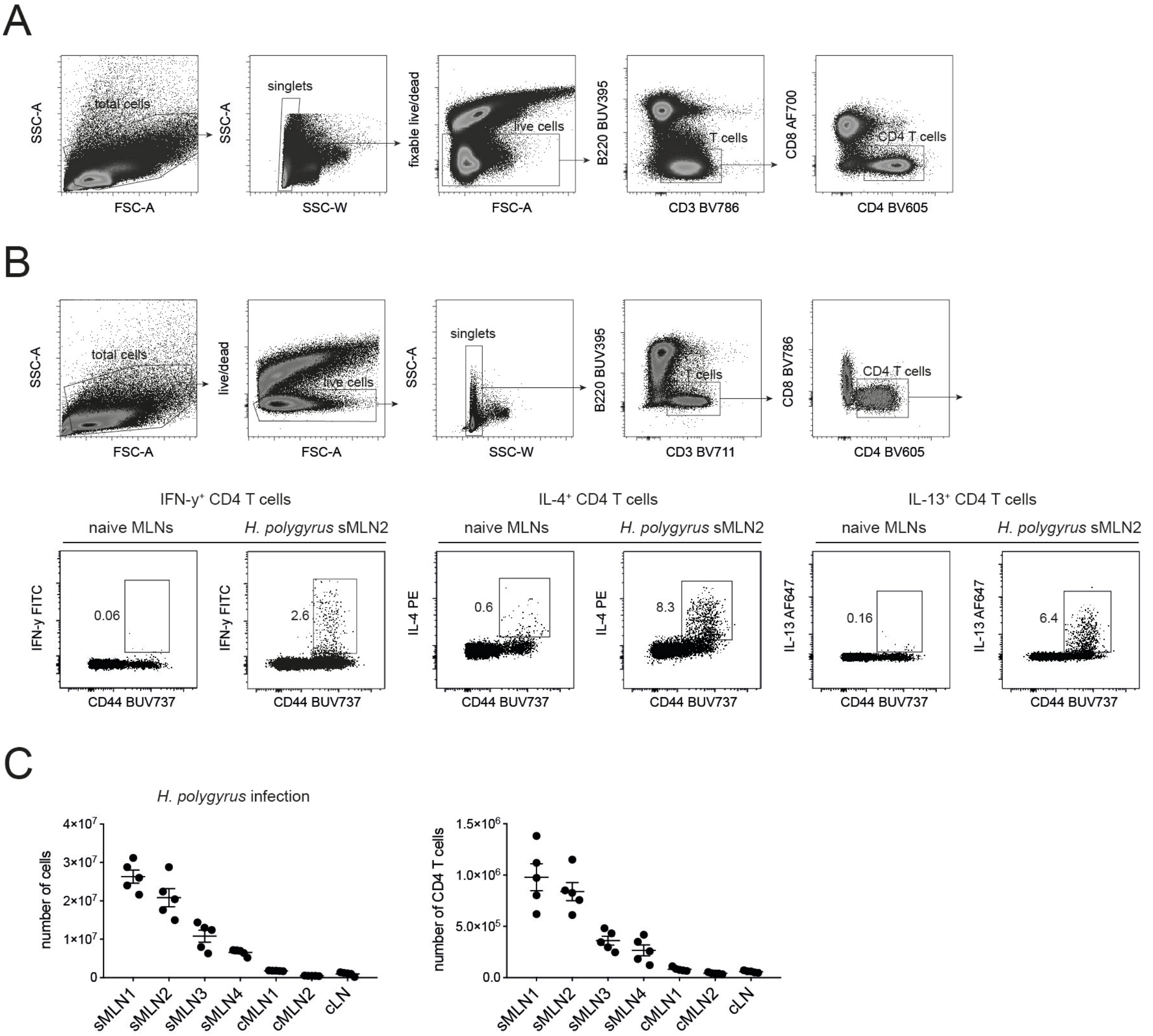

Supplementary Figure 2 (related to Main Figure 3). Assessment of T cell responses after *Heligmosomoides polygyrus* infection. (**A**) 20 µg of *H. polygyrus* ES antigen (HES) were injected in the footpad or ileal subserosa. Five days after immunization LNs were collected, stimulated with PMA/ionomycin for four hours and analysed by flow cytometry. IFN-γ and IL-4 producing CD4 T cells were identified by gating on viable single cells that expressed the T cell markers CD3 and CD4. (**B,C**) Mice were infected with 200 L3 *H. polygyrus* larvae by oral gavage and individual LNs were collected after 17 days, stimulated with PMA/ionomycin for four hours and analysed by flow cytometry. IFN-γ, IL-4 and IL-13 producing $CD4^+$ T cells were identified by gating on viable single cells that expressed the T cell markers CD3 and CD4. For infected mice, the number of total cells and CD4 T cells for each individual lymph node are shown in **C** (n=5 mice per group, representative of two independent experiments; mean±SEM).

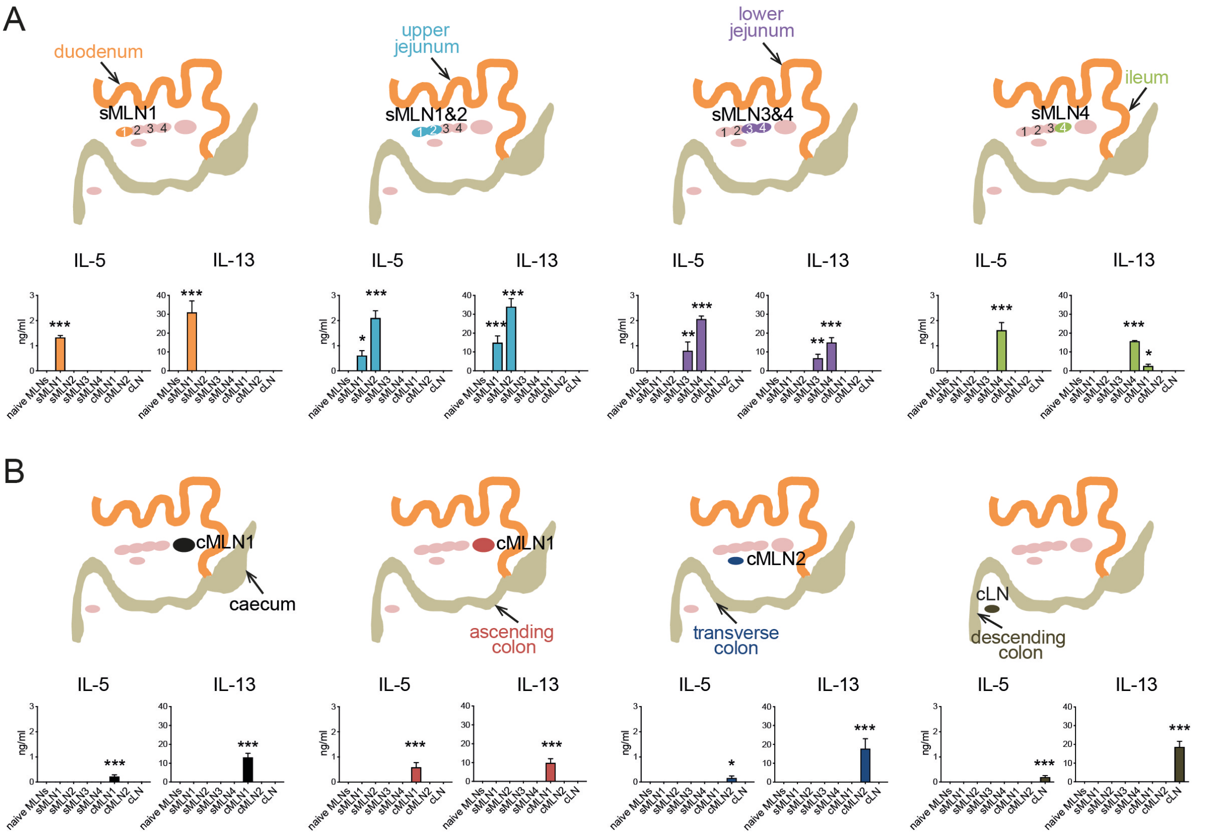

Supplementary Figure 3 (related to Main Figure 5). Subserosal injection of *Schistosoma mansoni* eggs induces segment specific IL-5 and IL-13 responses in the individual MLNs. (**A**) 1,000 *S. mansoni* eggs were injected into the different segments of the small intestine. Individual MLNs were collected five days after injection, LN cells were restimulated with SEA *in vitro* and IL-5 and IL-13 were measured by ELISA. Schematics indicate injection site and responding LN (n=3 mice per group, combined data from three independent experiments; mean±SEM; ordinary one-way ANOVA followed by Holm-Sidak's multiple comparisons test compare LN responses to naïve controls; *p≤0.05, **p≤0.01, ***p≤0.001). (**B**) Similar to **A**, 1,000 *Schistosoma mansoni* eggs were injected into the different segments of the large intestine. After 5 days LNs were collected, restimulated *in vitro*, and assessed for antigen specific cytokines by ELISA (n=3 mice per group, combined data from three independent experiments; mean±SEM; ordinary one-way ANOVA followed by Holm-Sidak's multiple comparisons test compare LN responses to naïve controls; *p≤0.05, ***p≤0.001).

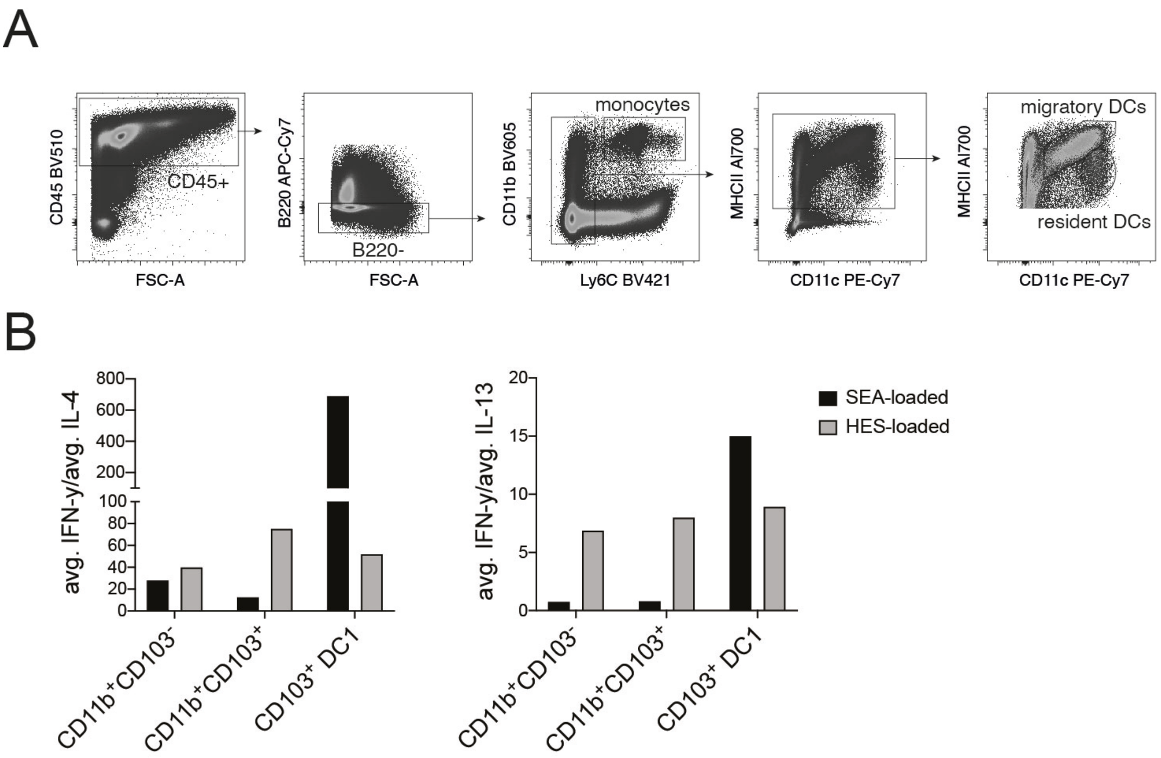

Supplementary Figure 4 (related to Main Figure 6). Identification of antigen presenting cells in the MLN and lymph. (**A**) 15 µg of AF660-labelled SEA was injected into the ileal subserosa. Pooled and individual MLNs were collected 24 hours after injection, enzymatically digested and analysed by flow cytometry. Different populations of antigen presenting cells were identified from live single cells that expressed CD45 and were $B220^-$. Monocytes were identified by their expression of Ly6C and CD11b, migratory dendritic cells were gated on $Ly6C^-$, $CD11c^+$ and $MHCII^{hi}$ cells, whereas resident dendritic cells expressed lower levels of MHCII. Lymph migratory dendritic cells were identified by the same strategy. (**B**) Ratio of average cytokine responses after transfer of SEA- or HES-loaded lymph DC subsets from data shown in **Figure 6 E** and **G**.
